# *Candida albicans* resistance to hypochlorous acid

**DOI:** 10.1128/mbio.02671-23

**Published:** 2023-11-30

**Authors:** Lois M. Douglas, Kyunghun Min, James B. Konopka

**Affiliations:** 1 Department of Microbiology and Immunology, Stony Brook University, Stony Brook, New York, USA; Duke University Hospital, Durham, North Carolina, USA

**Keywords:** *Candida albicans*, hypochlorous acid, bleach, fungal pathogen, stress resistance

## Abstract

Innate immune cells, especially neutrophils, play key roles in protecting against infection by *Candida albicans* and other fungal pathogens. A distinct aspect of neutrophils compared to other phagocytes is that they make much higher levels of myeloperoxidase, which converts H_2_O_2_ generated during the oxidative burst into highly reactive hypochlorous acid (HOCl). The effects of HOCl on *C. albicans* are not well defined, so comparative studies were used to show that HOCl kills *C. albicans* at lower doses than H_2_O_2_ and in a manner that correlates with permeabilization of the plasma membrane. Mutants with defects in plasma membrane organization and antioxidant pathways showed differential sensitivity to these oxidants, consistent with their distinct chemical properties. Transcriptional responses to HOCl and H_2_O_2_ were also quite different. Although they induced a common set of genes, most differentially regulated genes were unique. Testing the roles of genes that were predicted to be important for counteracting the types of thiol oxidation that are preferentially caused by HOCl showed that Mxr1 (methionine-S-sulfoxide reductase), Srx1 (sulfiredoxin that reduces cysteine-sulfinic acid groups), and Trx1 (thioredoxin that acts to reduce disulfide bonds) all promoted resistance to HOCl. Altogether, these results suggest that an effective way to help promote the killing of *C. albicans* by neutrophils would be to perturb the *C. albicans* plasma membrane, rather than targeting one specific antioxidant pathway.

**IMPORTANCE:**

Hypochlorous acid (HOCl), commonly known as bleach, is generated during the respiratory burst by phagocytes and is a key weapon used to attack *Candida albicans* and other microbial pathogens. However, the effects of hypochlorous acid on *C. albicans* have been less well studied than H_2_O_2_, a different type of oxidant produced by phagocytes. HOCl kills *C. albicans* more effectively than H_2_O_2_ and results in disruption of the plasma membrane. HOCl induced a very different transcriptional response than H_2_O_2_, and there were significant differences in the susceptibility of mutant strains of *C. albicans* to these oxidants. Altogether, these results indicate that HOCl has distinct effects on cells that could be targeted in novel therapeutic strategies to enhance the killing of *C. albicans* and other pathogens.

## INTRODUCTION

*Candida albicans* is a common fungal pathogen capable of growing in a wide range of niches in humans. *C. albicans* infections are an important concern because they can progress into lethal systemic infections, especially when immune defenses are compromised. This problem is exacerbated by the limited effectiveness of current antifungal drugs once a severe infection has been established (1). Thus, it is crucial to determine how *C. albicans* responds to the immune system in order to develop novel therapeutic strategies to enhance the host response to infection (2). Many different aspects of the immune system contribute to the defense against *C. albicans* (3, 4). However, innate immunity is key for counteracting *C. albicans* infections, which often progress rapidly (5, 6). Neutrophils play the most critical role, although macrophages and other types of innate immunity are also important. Neutrophils generate a strong respiratory burst that is a major weapon for attacking *C. albicans*, and they also form neutrophil extracellular traps that act on microbes that are too big to be phagocytosed, such as *C. albicans* hyphae or biofilms (7–9). The importance of neutrophils is also underscored by the fact that they represent ~60% of the cells in the blood, and neutropenic patients have increased susceptibility to infection by *C. albicans* and other microbial pathogens (10, 11).

The neutrophil respiratory burst initiates with the activation of NADPH oxidase to produce superoxide that is quickly converted to hydrogen peroxide (H_2_O_2_) (12, 13). In neutrophils, which are distinct from other types of innate immune cells by containing very high levels of myeloperoxidase, the H_2_O_2_ is acted on by myeloperoxidase to convert it into highly reactive hypochlorous acid (also known as HOCl or bleach) (13, 14). HOCl has chemically distinct properties from H_2_O_2_, and it is much more reactive. For example, a previous study found that it took about 10^8^ molecules of HOCl to kill *Escherichia coli*, whereas it took about 1,000 times more H_2_O_2_ (10^11^ molecules) (15). HOCl is also about 10 million-fold more reactive against thiols, especially thiols on cysteine and methionine (reviewed in reference 16). The cysteine sulfur group can be oxidized to form sulfenic acid, sulfinic acid, sulfonic acid, or a disulfide bond with another thiol group. Oxidation converts the methionine sulfur group to methionine sulfoxide or dehydromethionine (17). In addition, HOCl can chlorinate primary and secondary amines to convert them into chloramines, which can subsequently chlorinate and oxidize other molecules (18, 19). HOCl can, therefore, impact a wide range of macromolecules, including proteins, lipids, and nucleic acids. These features make HOCl very effective at killing microbes.

The ability of HOCl to act on *C. albicans* has been understudied relative to H_2_O_2_. This is likely due in part to previous assumptions that the effects of HOCl would be too broad for cells to have specific mechanisms to block its action (13, 20). However, recent studies with bacteria identified specific pathways that are activated to counteract the effects of HOCl, including chaperones that stabilize proteins that are misfolded after oxidation and antioxidant enzymes that reverse the oxidative damage to cysteine and methionine residues (17, 18, 21, 22). Therefore, to better understand how HOCl acts on *C. albicans*, we focused on three lines of experiments. First, we examined the ability of HOCl to attack the *C. albicans* plasma membrane (PM) and tested mutants with altered PM organization for susceptibility to HOCl. The PM is expected to be the first critical target HOCl encounters after it is created because HOCl is known to react very quickly. Another contributing factor could be that, in contrast to H_2_O_2_, which can cross membranes because it has only a small dipole moment and is therefore not very polar, about 50% of HOCl will be in the ionic hypochlorite form (ClO^−^) at the pH of the phagosome, which is not expected to cross the *C. albicans* PM (23). The second line of experiments assessed the *C. albicans* transcriptional response to HOCl, since this has not been reported previously and the response is expected to be different since HOCl reacts chemically in a very distinct way than H_2_O_2_. Third, we also assessed the sensitivity to HOCl of mutants lacking genes that encode antioxidant enzymes that can reduce oxidized sulfur groups on cysteine or methionine since they are predicted to contribute to reversing the damage caused by HOCl. The results define novel mechanisms for resisting HOCl, including a role for the PM. They also demonstrate that the genes that promote resistance to HOCl and H_2_O_2_ are often distinct. These results provide new insights into the mechanisms that promote the virulence of *C. albicans*.

## RESULTS

### Rapid PM permeabilization and killing of *C. albicans* by HOCl

**FIG 1:**
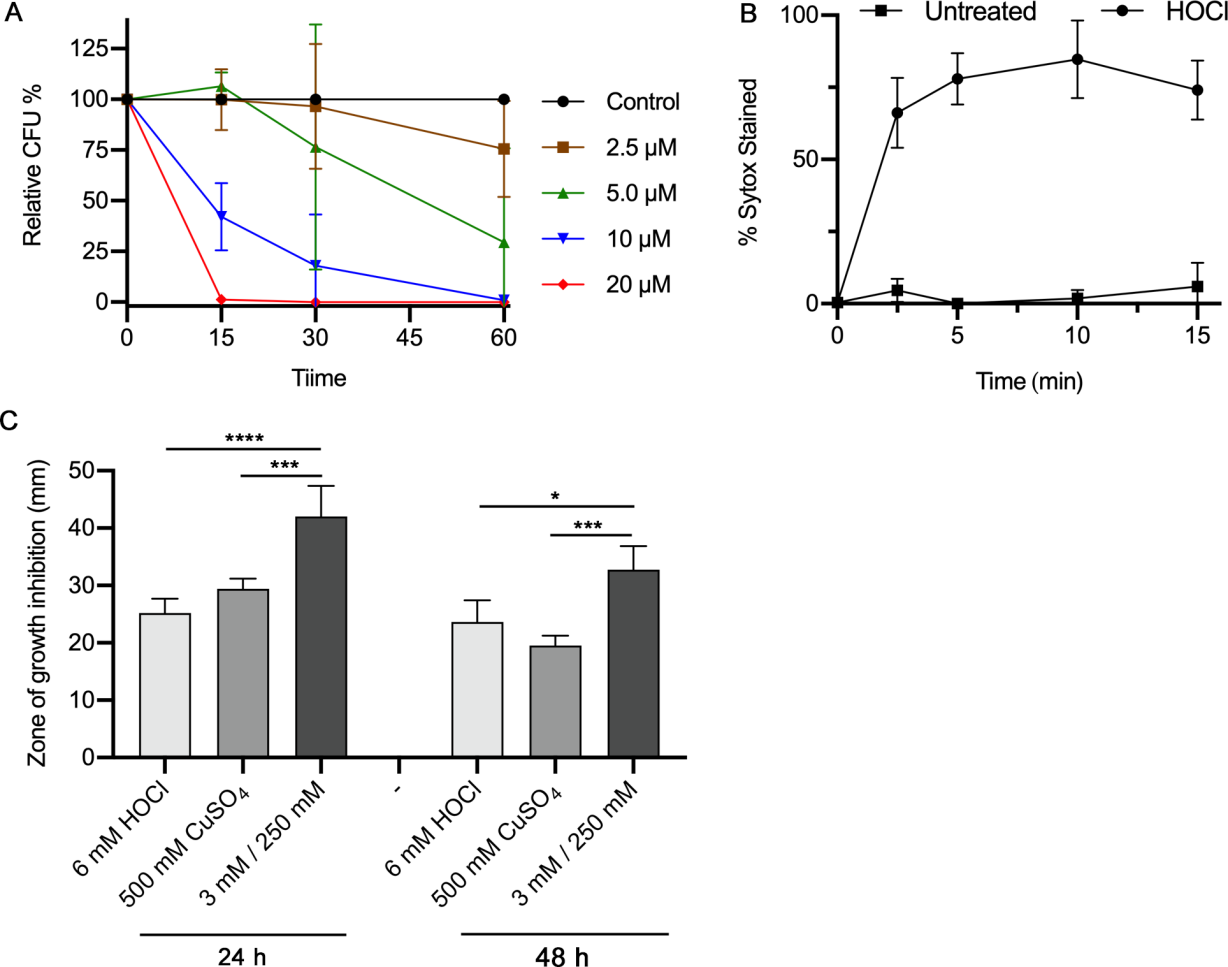
*C. albicans* killing and PM permeabilization in response to HOCl. (**A**) *C. albicans* cells (strain SC5314) were incubated with the indicated concentration of HOCl for the times indicated on the *x*-axis. The viable CFUs were determined by plating on agar medium. The results represent the average of four independent experiments. (**B**) *C. albicans* strain SC5314 was incubated with 20 µM HOCl for the time indicated on the *x*-axis and then stained with SYTOX Green, a membrane impermeable fluorescent dye that binds double-stranded nucleic acids.The results represent the average of three independent experiments. Error bars indicate SD. (**C**) The effects of a mixture of HOCl and CuSO_4_ were tested in diffusion assays, also known as halo assays. Then, 2.5 × 10^5^ SC5314 cells were spread onto the surface of a minimal medium plate, and then a 5-µL spot containing the indicated concentration of HOCl, CuSO_4_, or a mixture containing half the amount of HOCl and CuSO_4_ used in single compound assays was placed on the surface of the agar. The diameter of the zone of growth inhibition (halo) surrounding each spot was recorded after incubation for 24 or 48 h at 30°C. The results represent the average of four independent assays, each done in duplicate.

Time-course assays were carried out to define the doses and kinetics with which HOCl kills *C. albicans*. Cells were exposed to different concentrations of HOCl and then plated on agar medium to determine the viable colony-forming units (CFUs). (Note that dilutions of NaOCl were prepared, but since the pKa is ~7.4, there will be a mix of HOCl and ClO^−^. For simplicity, we will describe this mix as HOCl in the text and figures.) As shown in Fig. 1A, significant killing occurred at >10 µM HOCl. This is interesting since it is a much lower concentration than is needed for H_2_O_2_ to kill *C. albicans*, which is in the mM range (24). It was also interesting that HOCl acted very quickly. Treatment of cells for 15 min with 10 µM HOCl resulted in ~42% killing, and treatment with 20 µM HOCl resulted in ~99% killing. Additional loss of viability continued to occur at later time points through 60 min. In fact, there was even a significant drop in viability for cells treated for 60 min with 5 µM HOCl. These doses are expected to be in the range of HOCl concentrations that cells experience in the phagosome (19), although it has been challenging to estimate the concentration of HOCl in the phagosome because HOCl is short-lived due to its ability to rapidly react with cellular components.

We predicted that the PM would be a critical target for the highly reactive HOCl, as the PM would be an initial point of contact after HOCl is created in the phagosome. We therefore examined the integrity of the PM after HOCl treatment by staining cells with SYTOX Green, a membrane-impermeable fluorescent stain that binds nucleic acids (25). Interestingly, exposure of cells to 20 µM HOCl for as short as 2.5 min resulted in about 66% stained cells, indicating a high level of PM permeabilization (Fig. 1B). The percent of stained cells did not increase much with longer times of incubation. Some of our control studies indicated that this might be due to HOCl interfering with the SYTOX Green assay. Nonetheless, these studies show a very rapid permeabilization of the *C. albicans* PM. Damage to the PM is expected to exacerbate the effects of HOCl by facilitating the entry of HOCl and other oxidized products into the cytoplasm, where essential functions can be perturbed.

The ability of a combination of copper and HOCl to kill cells was examined since it has been shown that copper is pumped into the phagosome (26) and that copper is known to be redox-active and can attack the plasma membrane (27). Interestingly, a mix of CuSO_4_ and HOCl showed more substantial effects than either one alone, indicating a synergistic effect on cell killing (Fig. 1C).

### PM structure is important for resistance to oxidation

**FIG 2:**
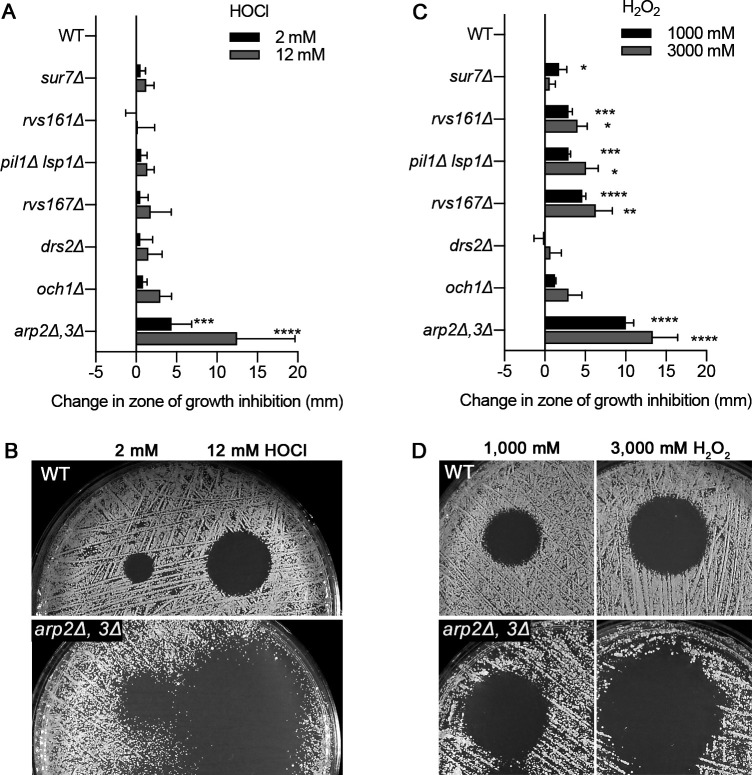
Susceptibility to HOCl of mutant strains with defects that alter PM function. The indicated strains were tested for sensitivity to (**A**) HOCl or (**C**) H_2_O_2_ in diffusion assays. For these assays, 2.5 × 10^5^ cells of the indicated strain were spread onto the surface of a minimal medium plate, and a 5 µL spot containing the indicated concentration of HOCl or H_2_O_2_ was placed on the surface of the agar. The diameter of the zone of growth inhibition surrounding each spot was recorded after incubation for 2 d at 30°C, and then the difference in size compared to the wild-type control cells is shown on the graphs. (**B and D**) Images of representative halo assays corresponding to strains with significant differences in HOCl sensitivity. The results represent the average of three independent assays, each done in duplicate. The strains used are described in Table 1.

The PM forms a critical barrier around cells that also participates in a wide range of dynamic functions essential for virulence, including secretion, endocytosis, morphogenesis, and cell wall synthesis (28). To better define how HOCl impacts PM function, we analyzed a set of mutants that were defective in endocytosis (*rvs161*Δ; *rvs167*Δ), MCC/eisosomes (*sur7*Δ; *pil1*Δ *lsp1*Δ), phospholipid flippase (*drs2*Δ), actin cytoskeleton (*arp2*Δ *arp3*Δ), and mannosyl transferase (*och1*Δ) that affects the PM and cell wall (27, 29–31) (Fig. 2). Disk diffusion (halo) assays, rather than assays in which cells are spotted onto an agar plate with a fixed concentration of a chemical, were used to test the mutants for sensitivity to the oxidants HOCl and H_2_O_2_ so that differences between strains could be quantified (Fig. 2A and B). The HOCl solution was spotted directly on the plate since we found that paper disks that are often used in disk-diffusion-type assays had unpredictable effects, with some disks quenching the effects of HOCl while others from the same batch had no effect. The results showed a trend in which all of the PM mutants showed larger zones of growth inhibition caused by HOCl, but only the *arp2*Δ *arp3*Δ strain reached statistical significance by analysis of variance (ANOVA; *P* < 0.0001). In contrast, the effects of H_2_O_2_ were often distinct from those of HOCl (Fig. 2B). For example, the *sur7*Δ, *pil1*Δ *lsp1*Δ*, rvs161*Δ, and *rvs167*Δ mutants all showed significantly increased susceptibility to H_2_O_2_.

### Susceptibility of antioxidant mutants to HOCl

**FIG 3:**
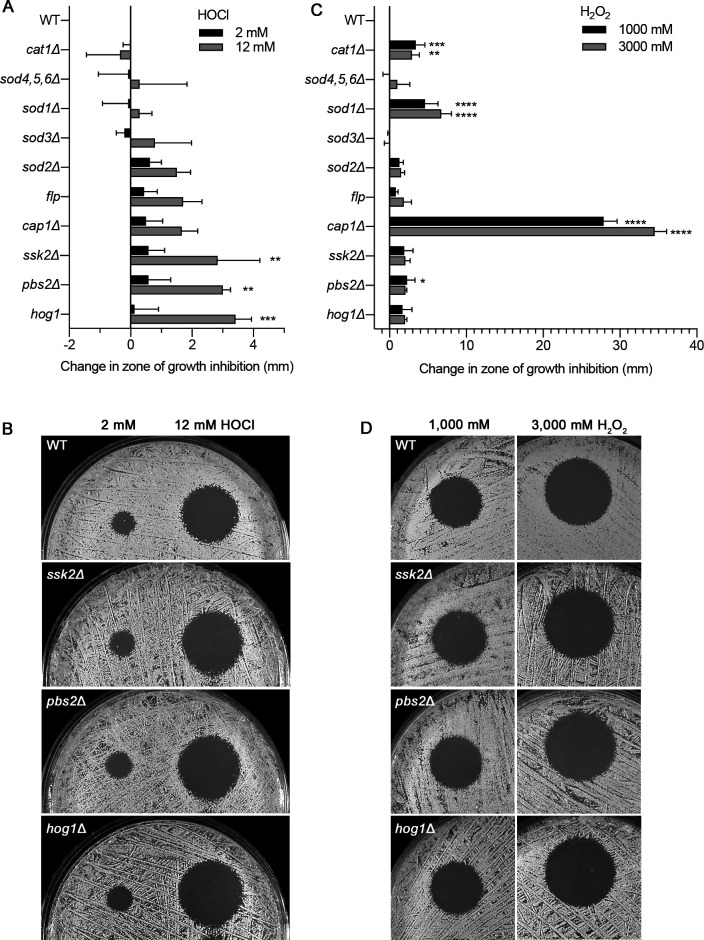
Susceptibility to HOCl of mutant strains with defects in antioxidant pathways. The indicated strains were tested for sensitivity to (**A**) HOCl or (**C**) H_2_O_2_ in agar plate diffusion assays as described in Fig. 2. The diameter of the zone of growth inhibition surrounding each spot was recorded after incubation for 2 days at 30°C, and then the difference in size compared to the wild-type control cells is shown on the graphs. (**B and D**) Images of representative halo assays corresponding to strains with significant differences in HOCl sensitivity. The results represent the average of three independent assays, each done in duplicate. The strains used are described in Table 1.

A set of mutants carrying mutations in known antioxidant enzymes was tested next for sensitivity to HOCl to determine whether any of these pathways play a critical role in resistance to HOCl (Fig. 3A). These mutants were defective in processes that included catalase, superoxide dismutases, flavodoxin-like proteins, the HOG MAP kinase pathway, and the Cap1 transcription factor that regulates the expression of many antioxidant genes (32–34). Although many of the mutants showed a trend toward increased susceptibility to HOCl, only the HOG pathway mutants *ssk2*Δ, *pbs2*Δ, and *hog1*Δ showed a statistically significant difference by ANOVA (*P* < 0.01). Interestingly, although these results identified a key role for the HOG map kinase pathway, only a limited effect at most was caused by the *cap1*Δ mutation.

Analysis of the susceptibility of these antioxidant mutants to H_2_O_2_ gave a very different pattern of results. As expected, the *cap1*Δ mutant showed very strong killing by H_2_O_2_ compared to the other strains, consistent with its role in regulating the expression of a broad range of antioxidant genes (Fig. 3B). In addition, the *cat1*Δ catalase mutant and *sod1*Δ superoxide dismutase mutants showed significantly increased susceptibility to H_2_O_2_ by ANOVA. In contrast, the HOG pathway mutants showed only a slight trend toward increased susceptibility to H_2_O_2_ that was only significant for the *pbs2*Δ mutant at one of the doses of H_2_O_2_ that were used (Fig. 3B). These results highlight the different effects HOCl and H_2_O_2_ have on cells and that they appear to be countered by different antioxidant mechanisms.

### RNA-seq analysis of HOCl-regulated genes

**FIG 4:**
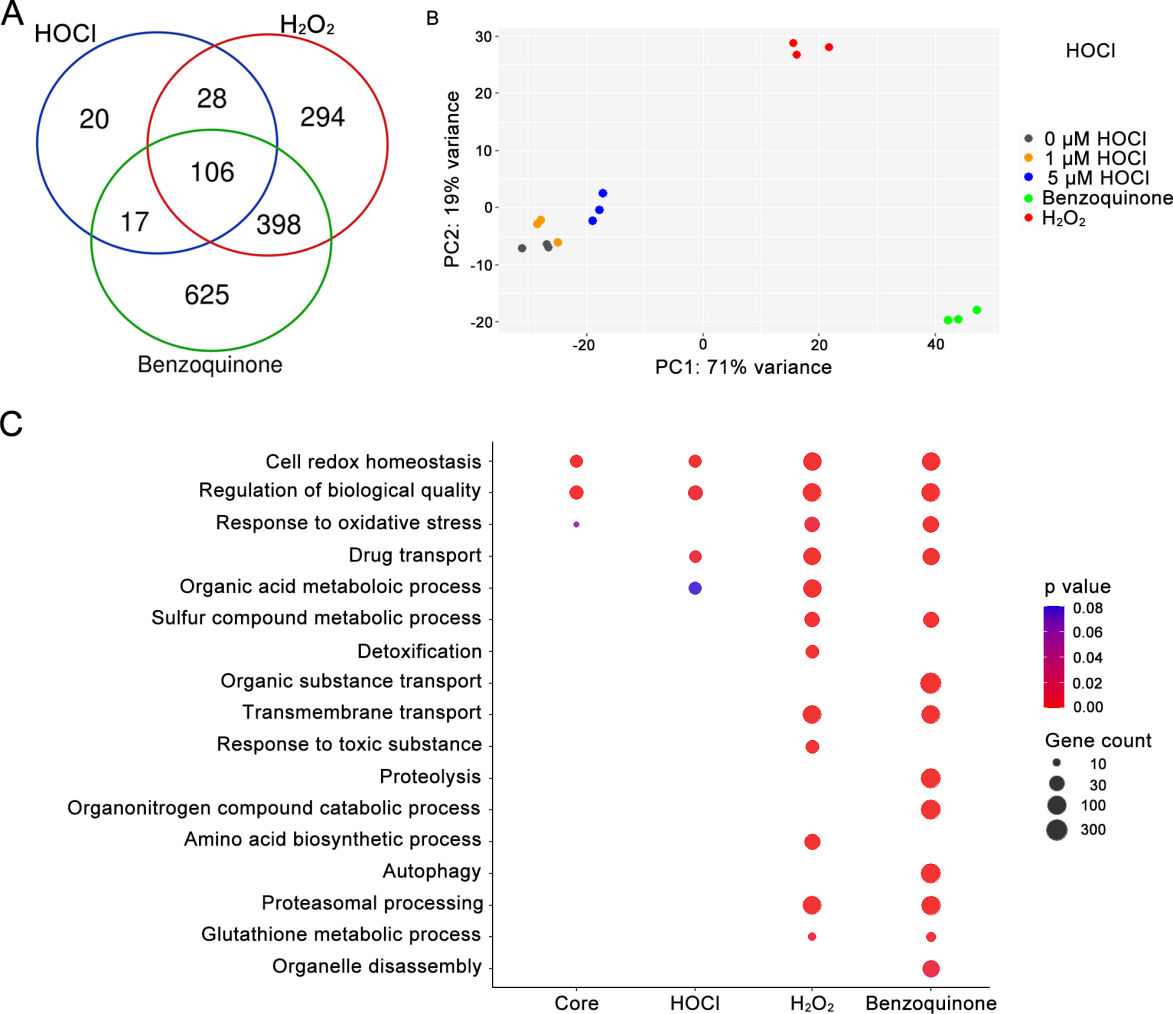
Transcriptomic analysis of *C. albicans* after exposure to HOCl, H_2_O_2_, or benzoquinone. (**A**) *C. albicans* WT cells were treated for 15 min with 5 µM HOCl, 5 µM H_2_O_2_, and 5 µM benzoquinone, respectively. The Venn diagram displays the number of upregulated genes (log_2_ fold change > 1, adjusted *P* value < 0.1). (**B**) Principal component analysis of the transcriptome. HOCl treatment samples (1 µM HOCl and 5 µM HOCl) clustered with the non-treatment control (0 µM HOCl). However, H_2_O_2_ and benzoquinone samples showed variable transcriptome patterns. Dots of the same color represent biological replicates. (**C**) Gene ontology (GO) term analysis of the upregulated genes under the indicated oxidative stress conditions. Core group genes were upregulated across all treatments, including HOCl, H_2_O_2_, and benzoquinone. Colors indicate statistical significance, and dot size represents the number of genes in the GO term.

To better understand how *C. albicans* responds to HOCl, we carried out RNA-seq analysis to identify the genes that are regulated by this oxidant. For comparison, we also analyzed the effects on transcription when cells were treated with H_2_O_2_ or with benzoquinone, which are chemically very different from HOCl. H_2_O_2_ is a peroxide that can oxidize a wide range of compounds, and benzoquinone can be converted to a semiquinone that generates reactive oxygen species (34). Cells were treated for 15 min with a sublethal dose of the oxidants that was determined to be the highest concentration that caused less than 1% reduction in CFUs. Interestingly, the patterns of gene regulation in response to these oxidants were very different (Fig. 4; Table S1). HOCl only induced 173 genes above the twofold cutoff, whereas H_2_O_2_ induced 826 genes and benzoquinone induced 1,146 genes. Only 106 genes were induced in common, although that represents more than half of the genes induced by HOCl. Principal component analysis revealed that the patterns of gene expression were also qualitatively very different. Transcriptomes from cells treated with sublethal doses of HOCl (1 or 5 µM) clustered very close to the untreated control cells, consistent with a small number of changes in gene expression (Table S1). In contrast, principal component analysis indicated that the patterns of gene expression affected by H_2_O_2_ and benzoquinone were very different from HOCl and from each other.

**FIG 5:**
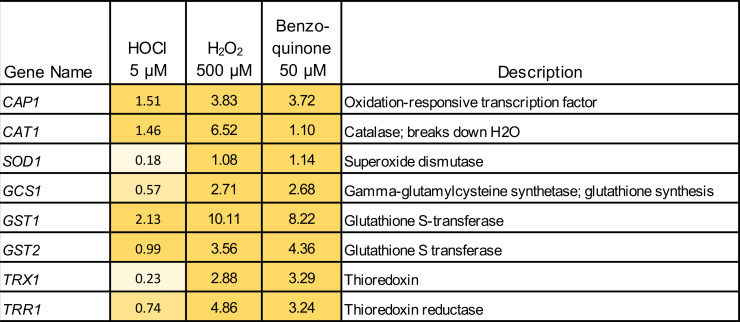
Expression of known antioxidant genes in response to different types of oxidative stress.The log_2_ fold induction of the indicated genes is shown for the condition indicated at the top. Shaded boxes indicate *P* values were significant (<0.05). The RNAseq data are shown in Table S1 and summarized in Fig. 4.

Gene ontology (GO) term analysis showed significant similarities in gene expression profiles regulated by all three oxidants for the categories of cell redox homeostasis, regulation of biological quality, and drug transport. These are consistent with cells counteracting oxidative stress, degrading damaged proteins, and pumping out toxic molecules. The gene expression profiles from cells treated with H_2_O_2_ and benzoquinone also showed similar GO term profiles for five other categories relating to response to oxidative stress and proteasomal processing. A comparison of a set of well-studied antioxidant genes, including those coding for catalase, superoxide dismutase, glutathione, and thioredoxin, showed a trend that they were induced by all three types of oxidative stress but were induced more weakly by HOCl (Fig. 5). Perhaps this contributes to the observed phenotype that a *cap1*Δ mutant was highly susceptible to H_2_O_2_ but not HOCl (Fig. 3).

**FIG 6:**
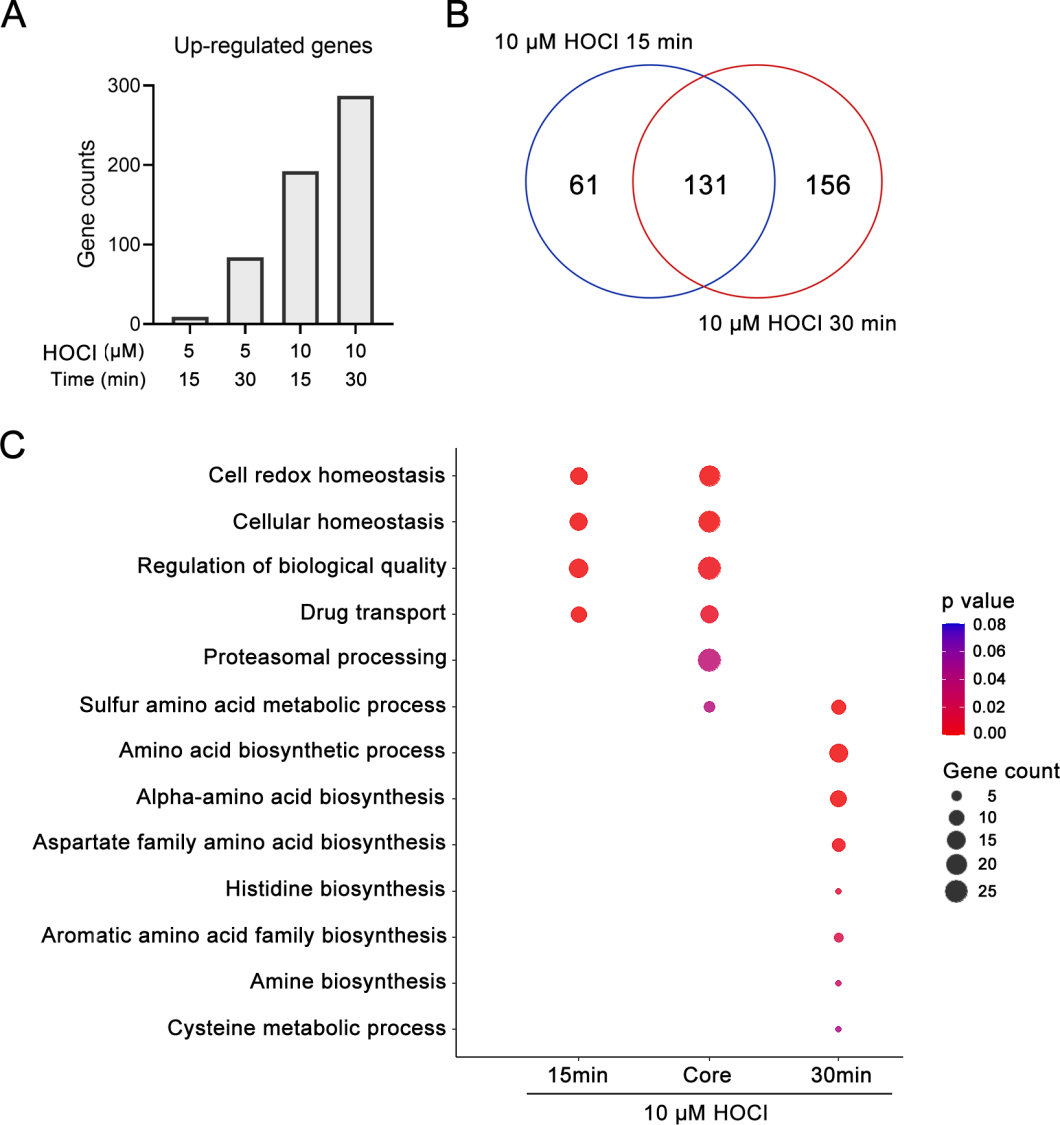
Changes in gene expression after different times of HOCl treatment. (**A**) *C. albicans* cells were treated with HOCl at various concentrations and for different durations. The plot displays the number of upregulated genes (log_2_ fold change > 1, adjusted *P* value < 0.1). (**B**) The Venn diagram shows the number of upregulated genes in 10 µM HOCl for 15 and 30 min, respectively (log_2_ fold change > 1, adjusted *P* value < 0.1). (**C**) GO term analysis of the upregulated genes in 10 µM HOCl for 15 and 30 min. Common group genes were upregulated in both 15- and 30-min treatments. Colors indicate statistical significance, and dot size represents the number of genes in the GO term.

The experiments described above examined *C. albicans* cells treated with 5 µM HOCl for 15 min. In view of the lower number of genes induced by HOCl compared to H_2_O_2_ and benzoquinone, we compared RNA-seq profiles from cells treated with 5 µM HOCl with those treated with a higher dose of HOCl (10 µM) after a 15 min incubation and a longer 30 min incubation. The results showed that both changes significantly increased the number of up-regulated genes (Fig. 6A). The strongest change was in cells treated with 10 µM HOCl for 30 min, which resulted in 287 upregulated genes.

There were 131 genes induced in common for cells incubated with 10 µM HOCl for 15 or 30 min (Fig. 6B). This represents 65% of the genes upregulated at 15 min and 46% of genes upregulated at 30 min. Analysis of the genes induced in common by GO term analysis showed that the major categories included response to oxidative stress and regulation of biological quality, consistent with cells attempting to deal with damage from HOCl. Genes specifically upregulated at 15 min were mapped to similar GO term categories, although the specific genes were different. In contrast, genes that were specifically induced at 30 min mapped to a distinct group of eight GO terms, all of which related to amino acid synthesis. This may be a reaction to help synthesize new proteins. It is also interesting to speculate that an increase in amino acids may have the beneficial effect of providing amine groups to react with HOCl, thereby protecting cellular proteins. Although the chloramines that would be created are still toxic, they are less so than HOCl.

### Antioxidant pathways that protect against HOCl

**FIG 7:**
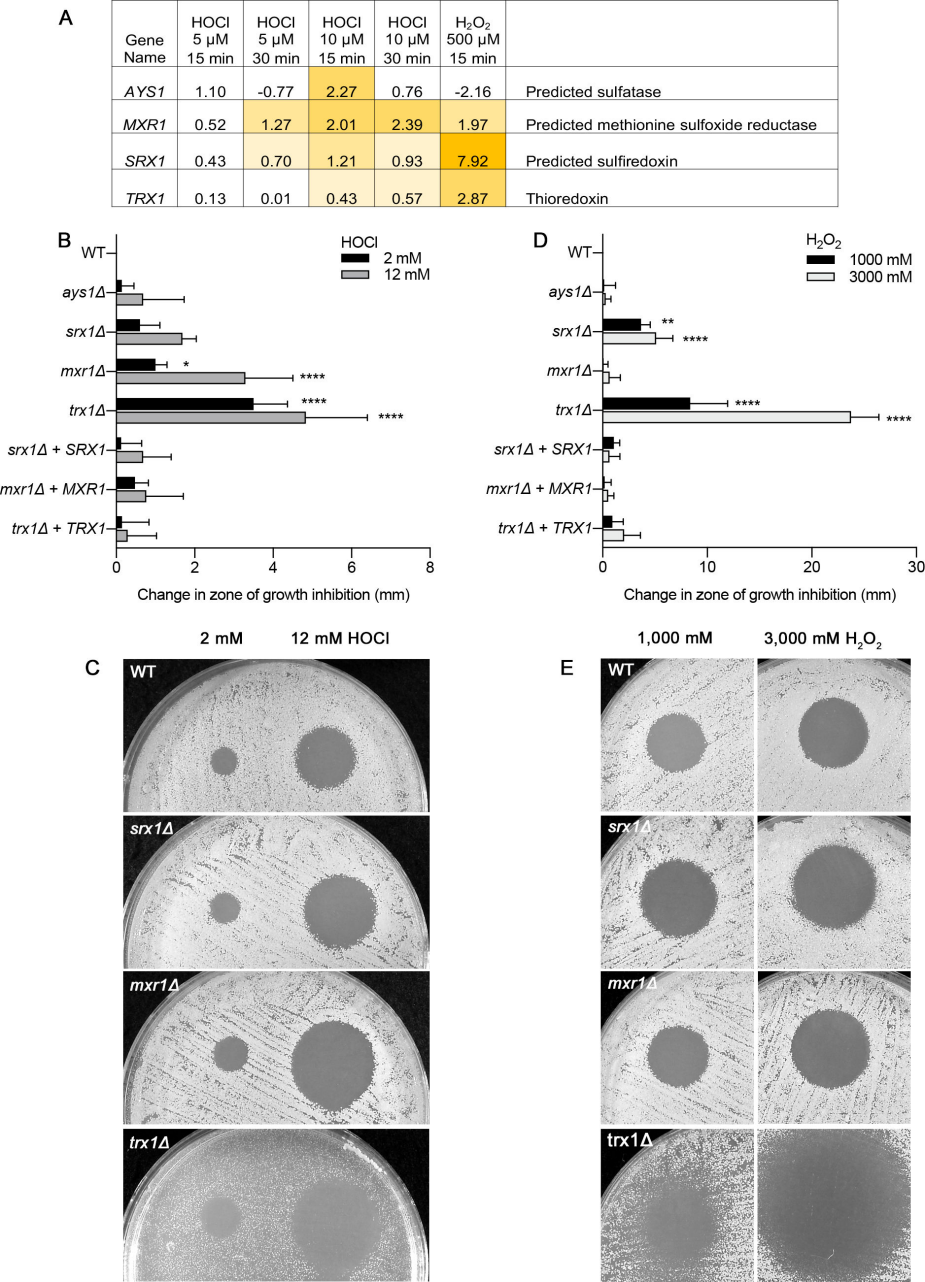
Susceptibility of *ays1*Δ, *mxr1*Δ, *srx1*Δ, and *trx1*Δ mutants to HOCl and H_2_O_2_. (A) Transcriptional regulation of and description of the roles of *AYS1*, *MXR1*, *SRX1*, and *TRX1* in reversing thiol oxidation. The log_2_ fold change in gene expression after treatment with different oxidants was determined by RNAseq. The data are shown in Table S1, and the data are summarized in Fig. 4 and 5. Shaded boxes indicate significant *P* values. (**B and D**) Susceptibility of the *ays1*Δ, *mxr1*Δ, *srx1*Δ, and *trx1*Δ mutants to (**B**) HOCl and (**D**) H_2_O_2_. The *x*-axis reports the change in diameter of the zone of growth inhibition caused by spotting 5 µL of the indicated concentration of HOCl on a lawn of the indicated type of *C. albicans* cells. (**C and E**) Images of representative halo assays for the data shown in panels B and D. The results represent the average of three independent experiments, each done in duplicate. The *trx1*Δ strain grows poorly in minimal medium because Trx1 is also needed for methionine synthesis (37). Error bars indicate SD.

Inspection of the genes induced by HOCl revealed the presence of four understudied genes that are implicated in reversing damage caused by HOCl (Fig. 7A). Two genes, *MXR1* (C2_00,960C; orf19.2028) and *SRX1* (C2_05,060C; orf19.3537), function to reverse oxidative damage to sulfur-containing amino acids that are a major target of HOCl. *MXR1* encodes methionine-S-sulfoxide reductase, and *SRX1* encodes a sulfiredoxin that reduces cysteine-sulfinic acid groups. The other two genes include *AYS1* (C3_02,360C; orf19.1608), which encodes an enzyme with similarity to arylsulfitases that cleave sulfate esters, and *TRX1* (CR_10,350C; orf19.7611), which encodes thioredoxin, a key antioxidant in eukaryotic cells. All of these genes are broadly conserved, although *AYS1* is absent from species related to *Saccharomyces cerevisiae* and *Candida glabrata*. Similar results were reported previously for the induction of *C. albicans* genes by H_2_O_2_ (35) and the *MXR1*, *SRX1, and AYS1* genes were reported to be induced after exposure to neutrophils (36).

Testing the killing by HOCl revealed that the *ays1*Δ, *srx1*Δ, *mxr1*Δ, and *trx1*Δ mutants all displayed a trend toward increased susceptibility to HOCl, although this only reached statistical significance by ANOVA for the *mxr1*Δ and *trx1*Δ mutants (Fig. 7B). Once again, the pattern for H_2_O_2_ was distinct from HOCl. Only *trx1*Δ showed significantly increased susceptibility to both H_2_O_2_ and HOCl. Interestingly, the *srx1*Δ mutant was significantly more susceptible to H_2_O_2_ but not HOCl, whereas the opposite was true for the *mxr1*Δ mutant. The *ays1*Δ did not show a significant increase in susceptibility to H_2_O_2_, although it did show a trend toward increased killing by HOCl. Thus, although *MXR1*, *SRX1*, and *TRX1* all promote resistance to oxidation, they have differential effects in resisting HOCl and H_2_O_2_.

## DISCUSSION

The respiratory burst by neutrophils is a key aspect of innate immunity that helps to prevent systemic infections by *C. albicans* and many other microbial pathogens. A distinctive feature of neutrophils compared to other phagocytes is that they make much higher levels of myeloperoxidase and therefore more HOCl (13). HOCl has distinct chemical properties compared to H_2_O_2_ that make it an advantageous addition to the neutrophil arsenal. In particular, it reacts more quickly than H_2_O_2_. In addition, about 50% will be in the ionic hypochlorite form (ClO^−^) in the phagosome. Both of these properties make it less likely that it will diffuse across the phagosomal membrane. In contrast, H_2_O_2_ is able to diffuse across the phagosomal membrane and damage the cytoplasmic components of neutrophils. HOCl also causes different types of oxidative damage than H_2_O_2_, such as oxidation of sulfur groups on proteins and chlorination of amine groups (7, 13, 16–18). Therefore, the goals of this study were to better define how *C. albicans* responds to HOCl.

HOCl killed *C. albicans* quickly and at relatively low µM doses *in vitro*, which are about 500-fold lower than a lethal dose of H_2_O_2_ (Fig. 1A). The concentrations of HOCl that kill *C. albicans* appear to be in the range that is generated in the phagosome, although it is difficult to compare *in vitro* studies and the phagosome because the levels of HOCl are dynamic (19, 38). The rapid increase in HOCl during the respiratory burst is balanced by its ability to react quickly with other molecules. Also, our *in vitro* studies used a single addition of HOCl rather than a sustained burst of HOCl synthesis that occurs in the phagosome. Other considerations include the fact that there is not much extra space in the lumen of the phagosome surrounding *C. albicans*, and it has been reported that myeloperoxidase may attach to microbes to help target the HOCl more effectively (39). Interestingly, humans with myeloperoxidase deficiency are reported to be slightly more susceptible to *C. albicans* infection, although the majority are thought to be asymptomatic (10). However, myeloperoxidase deficiency is associated with a greater risk of infection by *C. albicans* in patients with other underlying diseases such as diabetes (40). The observation that mpo^−^/mpo^−^ mice that lack myeloperoxidase are more susceptible to *C. albicans* also supports a significant role for HOCl (41).

The effects of HOCl on the PM were examined because it was expected to be the first critical target encountered by HOCl. Consistent with this, HOCl killed *C. albicans* quickly in a manner that coincided with the permeabilization of the PM (Fig. 1B). Furthermore, a variety of mutants with altered PM function showed a trend toward increased susceptibility to HOCl, with an *arp2*Δ *arp3*Δ mutant having the strongest phenotype (Fig. 2A). The *arp2*Δ *arp3*Δ mutant was also more susceptible to H_2_O_2_ (Fig. 2B) and to copper (27), which is pumped into the phagosome of macrophages and likely neutrophils (26). The Arp2/3 complex promotes branching of actin filaments, which has been shown to strengthen the PM in other organisms (42). This suggests that the actin cytoskeleton helps stabilize the PM after it is damaged by HOCl and other agents found in the phagosome. Studies with *S. cerevisiae* suggested that exposure to HOCl can promote cell death by apoptosis, but these studies used a long 16-h exposure to HOCl (43). In contrast, our studies indicate that the rapid permeabilization of the PM is a key underlying event, as it will also allow HOCl and ClO^−^ to enter the cytoplasm and cause greater oxidative damage.

Testing of mutant strains with defects in known antioxidant pathways revealed a trend suggesting that several different functions contribute to resisting the effects of HOCl, but only the HOG MAP kinase pathway mutants reached statistical significance (Fig. 3). In contrast, the HOG pathway played at most a minor role in resisting H_2_O_2_, as the changes in susceptibility were generally not statistically significant. The identification of a role for the HOG pathway is consistent with HOCl causing PM damage since the HOG pathway is known to respond to cell wall and PM stress (44). Interestingly, the Cap1 transcription factor did not play a significant role in promoting resistance to HOCl, whereas it is very important for resisting H_2_O_2_. This could be because HOCl was a much weaker inducer of Cap1-regulated antioxidant genes (Fig. 5). Perhaps *C. albicans* does not ordinarily encounter HOCl as a commensal in the gastrointestinal tract and has therefore not evolved more effective mechanisms to counteract the rapid effects of HOCl.

Transcriptomic studies showed that *C. albicans* responds very differently to HOCl compared to two other oxidants: H_2_O_2_ and benzoquinone (Fig. 4). This is consistent with their different chemical properties and suggests that there is a limited core stress response induced by these different oxidants. An interesting aspect of the RNA-seq studies was the difference between cells treated with HOCl for 15 min versus 30 min. At 15 min, the major GO terms associated with the induced genes related to oxidative stress responses (Fig. 6). However, at 30 min, the major GO terms relate to amino acid synthesis. One possibility is that this reflects new protein synthesis to replace damaged proteins. Another interesting possibility is that increasing the pool of amino acids would protect cells by providing substrates to react with HOCl and prevent it from causing further cellular damage.

The defense against HOCl was examined further by studying four genes (*AYS1*, *SRX1*, *MXR1*, and *TRX1*) that were predicted to play a role in protecting against the types of oxidative damage caused by HOCl (Fig. 7A). The corresponding deletion mutant cells showed a trend toward increased susceptibility, but only the *mxr1*Δ and *trx1*Δ mutants displayed statistically significant increased susceptibility to HOCl by ANOVA (Fig. 7). This is consistent with their predicted functions, since Mxr1 is similar to methionine-S-sulfoxide reductases and the Trx1 thioredoxin acts to reduce disulfide bonds. Interestingly, the *srx1*Δ mutant, which lacks a protein that is similar to sulfiredoxins that reduce cysteine-sulfinic acid groups, displayed significantly increased susceptibility to H_2_O_2_, whereas the *mxr1*Δ mutant did not.

Altogether, the results of this study demonstrate that there are key differences in the ways that HOCl and H_2_O_2_ attack *C. albicans*. This makes it important for *C. albicans* to utilize a broad range of different ways to resist oxidative stress, which is also important because there is interconversion between different ROS species. In addition, there can be synergistic effects, such as the combined effects of copper and HOCl (Fig. 1C). Given the variety and redundancy of antioxidant mechanisms in *C. albicans*, our studies indicate that efforts to design novel therapeutic strategies to enhance the killing of *C. albicans* by neutrophils may benefit from alternative strategies, such as perturbing PM function, rather than targeting a specific antioxidant pathway.

## MATERIALS AND METHODS

### Strains and media

The genotypes of the *C. albicans* strains used are described in Table 1. Cells were grown in rich YPD medium (2% yeast extract, 1% peptone, 2% dextrose, 80 mg/L uridine) or a synthetic medium containing yeast nitrogen base, 2% dextrose, amino acids, and uridine if necessary (45).

**TABLE 1:** *C. albicans* strains used in this study

Strain	Reference	Short genotype	Full genotype
BWP17	(46)	Parental strain	*his1*::*hisG*/*his1*::*hisG arg4*::*hisG*/*arg4*::*hisG ura3*::λ*imm434*/*ura*3::λ*imm434*
DIC185	(46)	Prototrophic WT control	*ura3*::*λimm434*/*URA3 his1*::*hisG*/*HIS1 arg4*::*hisG*/*ARG4*
SN152	(47)	Parental strain	*arg4*∆/*arg4*∆ *leu2*∆/*leu2*∆ *his1*∆/*his1*∆ *URA3*/*ura3∆::imm434 IRO1*/*iro1*∆::*imm434*
YLD233-1	This study	Prototrophic WT control	*ARG4*/*arg4*∆ *leu2*∆/*leu2*∆::*CmLEU2 his1*∆/*his1*∆::*CdHIS1*
SC5314	(48)	Clinical isolate	
YJA11	(49)	*sur7*Δ	*sur7*Δ::*ARG4*/*sur7*Δ::*HIS1 URA3*/*ura3*::λ*imm434 his1*::*hisG*/*his1*::*hisG arg4*::*hisG*/*arg4*::*hisG*
YHXW21-1	(30)	*pil1*Δ *lsp1*Δ	*pil1*Δ::*ARG4*/*pil1*Δ::*FRT lsp1*Δ::*HIS1*/*lsp1*Δ::*SAT1 flipper URA3*/*ura3*::λ*imm434 his1*::*hisG*/*his1*::*hisG arg4*::*hisG*/*arg4*::*hisG*
LLF60A	(34)	*pst1*Δ *pst2*Δ *pst3*Δ *ycp4*Δ	*pst3-ycp4*Δ::*LEU2*/*pst3-ycp4*Δ::*HIS1pst2*Δ::*FRT*/*pst2*Δ*::FRT pst1*Δ::*FRT*/*pst1*Δ::*FRT ARG4*/*arg4*Δ
YLD197-1	(50)	*pbs2*	*pbs2*Δ::*HIS1*/*pbs2*Δ::*LEU2 his1*Δ/*his1*Δ *leu2*Δ/*leu2*Δ *ARG4*/*arg4*Δ *URA3*/*ura3*::*imm IRO1*/*iro1*Δ::i*mm*
YLD185-7	(50)	*ssk2*Δ	*ssk2*Δ::*HIS1*/*ssk2*Δ::*LEU2 his1*Δ/*his1*Δ *leu2*Δ/*leu2*Δ *ARG4*/*arg4*Δ *URA3*/*ura3*::*imm IRO1*/*iro1*Δ::*imm*
YLD184-3	(50)	*hog1*Δ	*hog1*Δ::*HIS1*/*hog1*Δ::*LEU2 his1*Δ/*his1*Δ *leu2*Δ/*leu2*Δ *ARG4*/*arg4*Δ *URA3*/*ura3*::*imm IRO1*/*iro1*Δ::*imm*
YLD14-3	(29)	*rvs161*Δ	*rvs161*Δ::*ARG4*/*rvs161*Δ::*HIS1 URA3*/*ura3*::λ*imm434 his1*::*hisG*/*his1*::*hisG arg4*::*hisG*/*arg4*::*hisG*
YLD16-11	(29)	*rvs167*Δ	*rvs167*Δ::*ARG4*/*rvs167*Δ::*HIS1 URA3*/*ura3*::λ*imm434 his1*::*hisG*/*his1*::*hisG arg4*::*hisG*/*arg4*::*hisG*
YLD188-1	(50)	*och1*	*och1*Δ::*HIS1*/*och1*Δ::*LEU2 his1*Δ/*his1*Δ *leu2*Δ/*leu2*Δ *ARG4*/*arg4*Δ *URA3*/*ura3*::*imm IRO1*/*iro1*Δ::*imm*
MT505-A	(51)	*cat1*Δ	*cat1*Δ::FRT/*cat1*Δ::FRT
CaEE227	(31)	*arp2*Δ *arp3*Δ	*arp2*::*LEU2*/*arp2*::*HIS1 arp3*::*URA arp3*::*ARG4*
YLD220-14-18-1	(27)	*drs2*Δ	*drs2*Δ::*ARG4/drs2*Δ::*HIS1 URA3*/*ura3*::λ*imm434 his1*::*hisG*/*his1*::*hisG arg4*::*hisG*/*arg4*::*hisG*
YLD224-9	(27)	*neo1*Δ	*neo1*Δ::*HIS1/neo1*Δ::*LEU2 HIS1*/*his1*Δ *leu2*Δ/*leu2*Δ *ARG4*/*arg4 URA3*/*ura3*::*imm IRO1*/*iro1*Δ::*imm*
YLD240-8–2	This study	*trx1*∆	*trx1*∆::*LEU2*/*trx1*∆::*LEU2 ARG4*/*arg4*∆ *leu2*∆*leu2*∆ *HIS1*/*his1*∆ *URA3*/*ura3*∆::*imm434 IRO1*/*iro1*∆::*imm434*
YLD259-8-2-1	This study	*trx1*∆*TRX1*	*trx1*∆::*LEU2*/*trx1*∆::*LEU2 ARG4*/*arg4*∆ *leu2*∆*leu2*∆ *HIS1*/*his1*∆ *URA3*/*ura3*∆::*imm434 IRO1*/*iro1*∆::*imm434 NEUT5L*/*neut5l*::*TRX1-NAT1*
YLD253-10-6-3	This study	*mxr1*∆	*mxr1*∆::*LEU2*/*mxr1*∆::*LEU2 ARG4*/*arg4*∆ *leu2*∆*leu2*∆ *HIS1*/*his1*∆ *URA3*/*ura3*∆::*imm434 IRO1*/*iro1*∆::*imm434*
YLD14-2-1-6	This study	*mxr1*∆ *MXR1*	*mxr1*∆::*LEU2*/*mxr1*∆::*LEU2 ARG4*/*arg4*∆ *leu2*∆*leu2*∆ *HIS1*/*his1*∆ *URA3*/*ura3∆::imm434 IRO1/iro1∆*::*imm434 NEUT5L*/*neut5l*::*MXR1-NAT1*
YLD246-11-2-2	This study	*srx1*∆	*srx1*∆::*LEU2*/*srx1*∆::*LEU2 ARG4*/*arg4*∆ *leu2*∆*leu2*∆ *HIS1*/*his1*∆ *URA3*/*ura3*∆::*imm434 IRO1*/*iro1*∆::*imm434*
YLD257-5-1-4-2	This study	*srx1*∆ *SRX1*	*srx1∆::LEU2*/*srx1*∆::*LEU2 ARG4*/*arg4*∆ *leu2*∆*leu2*∆ *HIS1*/*his1*∆ *URA3*/*ura3*∆::*imm434 IRO1*/*iro1*∆::*imm434 NEUT5L*/*neut5l*::*SRX1-NAT1*
YLD252-8-1-9	This study	*ays1*∆	*ays1*∆::*LEU2*/*ays1*∆::*LEU2 ARG4*/*arg4*∆ *leu2*∆*leu2*∆ *HIS1*/*his1*∆ *URA3*/*ura3*∆::*imm434 IRO1*/*iro1*∆::*imm434*
YLD236-3	(51)	*sod1*∆	*sod1*∆::*CmLEU2*/*sod1*∆::*CdHIS1 ARG4*/*arg4*∆ *leu2*∆/*leu2*∆ *his1*∆/*his1*∆ *URA3*/*ura3*∆::*imm434 IRO1*/*iro1*∆::*imm434*
YLD237-4	(51)	*sod2*∆	*sod2*∆::*CmLEU2*/*sod2*∆::*CdHIS1 ARG4*/*arg4*∆ *leu2*∆/*leu2*∆ *his1*∆/*his1*∆ *URA3*/*ura3*∆::*imm434 IRO1*/*iro1*∆::*imm434*
YLD238-1	(51)	*sod3*∆	*sod3*∆::*CmLEU2*/*sod3*∆::*CdHIS1 ARG4*/*arg4*∆ *leu2*∆/*leu2*∆ *his1*∆/*his1*∆ *URA3/ura3*∆::*imm434 IRO1*/*iro1*∆::*imm434*
YLD239-1	(51)	*sod5*∆*sod4*∆ *sod6*∆	*sod5*∆::*CmLEU2*/*sod5*∆::*CdHIS1 sod4*∆::*FRT*/*sod4*∆::*FRT sod6*∆::*FRT*/*sod6*∆::*FRT ARG4*/*arg4*∆ *leu2*∆*/leu2*∆ *his1*∆/*his1*∆*URA3*/*ura3*∆::*imm434 IRO1*/*iro1*∆::*imm434*
YLD260-4	(50)	*cap1*∆	*cap1*∆::*CmLEU2*/*cap1*∆::*CdHIS1 ARG4*/*arg4*∆ *leu2*∆/*leu2*∆ *his1*∆/*his1*∆ *URA3*/*ura3*∆*::imm434 IRO1*/*iro1*∆::*imm434*

Homozygous deletion mutants lacking the *AYS1*, *MXR1*, *SRX1*, and *TRX1* genes were constructed using transient expression of CRISPR-Cas9 in *C. albicans* strain SN152 (47), essentially as described previously (52, 53). Cassettes for *CaCAS9* expression, single guide RNA (sgRNA) expression, and a repair template with the selectable marker were co-transformed into cells. The Ca*CAS9* gene was codon optimized for expression in *C. albicans* (54). The *CaCAS9* expression cassette, which was codon optimized for expression in *C. albicans*, was PCR amplified from the plasmid pV1093 (kindly provided by Dr. Valmik Vyas) (54). The cassettes for the sgRNA expression were constructed by PCR using the plasmid template pV1093 and 20-bp target sequences for each gene that were defined previously by Vyas et al. (55). The sgRNA was used to target Cas9 to make a DNA double-strand break at specific target sites (Table S2) (52). Repair templates were constructed by PCR using primers with ~80 bases of homology to the sequences upstream or downstream from the target region to amplify Cm*LEU2* on plasmid pSN40 (47). The oligonucleotide primers are listed in Table S2. PCR was conducted with Ex Taq polymerase (TaKaRa Bio, Inc.). PCR products were purified by extraction with a phenol/chloroform/isoamyl alcohol mixture (25:24:1). DNA was introduced into cells by the lithium acetate method (56). Homozygous deletion mutants were identified by PCR amplification of genomic DNA using primers that flanked the 5′ and 3′ ends of the genes as well as internal primers. Four independent isolates for each mutant were examined to verify that they displayed the same phenotype. To generate complemented strains, the corresponding wild-type gene containing 500 bp upstream and 350 bp downstream was PCR amplified and then inserted into *Sma* I-cleaved pDIS3 by gap repair in *S. cerevisiae* strain W3031A, as described previously (57). The wild-type gene was amplified using primers containing 80 bp of homology to the ends of *Sma* I-digested pDIS3 to facilitate the gap repair. The resulting plasmid was digested with *Sfi* I to release the wild-type gene and the *NAT1* selectable marker, flanked by sequences corresponding to the NEUT5L locus, and then transformed into the *C. albicans* deletion strain to integrate the wild-type gene at NEUT5L. The oligonucleotides used to construct the *C. albicans* strains are described in Table S2.

### Assays for killing by HOCl and other oxidants

For the assessment of cell viability by CFU assay following incubation in HOCl, an overnight dilution series was set up in minimal medium and incubated at 30°C with rotation. The next day, log-phase cells were washed twice and resuspended in sterile deionized H_2_O. Cells were diluted to a final concentration of 1 × 10^6^ cells/ml in a reaction volume of 1 ml containing 1 mM sodium phosphate pH 7.4 buffer and 0, 2.5, 10, or 20 μM HOCl. Following incubation for 15, 30, or 60 min at 30°C with rotation, 30 μL of reaction was added to 10 ml of sterile deionized H_2_O, and then 100 μL was spread onto YPD agar plates. After incubation for 48 h at 30°C, colony-forming units were counted.

SYTOX Green (Invitrogen, Molecular Probes, Eugene, OR, USA) is a membrane-impermeable nucleic acid stain that can be used to assay plasma membrane integrity (25). For the analysis, cells were grown in synthetic medium overnight at 30°C to log phase, washed in sterile H_2_O, and diluted to 1 × 10^6^ cells/ml. Following incubation in 20 µM HOCl at 30°C for the indicated time, 500 μM methionine was added to a final concentration of 45 μM to quench the reaction. The cells were washed in sterile H_2_O, SYTOX Green was added to a final concentration of 2.5 nM, and the cells were incubated at room temperature for 5 min. Cells were washed again in sterile H_2_O and then analyzed by fluorescence microscopy. Images were obtained using an Olympus BH2 microscope equipped with a Zeiss AxioCam digital camera. The percent of stained cells was determined by counting 50–200 cells in three independent experiments.

Halo assays used to quantify the sensitivity of *C. albicans* cells to HOCl and other oxidants were carried out with strains that were grown overnight in YPD medium at 30°C with rotation. The cells were harvested by centrifugation, resuspended in sterile H_2_O at a density of 1.0 × 10^6^ cells/ml, and then 250 μL was spread onto the surface of a synthetic medium agar plate. After allowing the cell mixture to dry on the plate, 5 µL of the indicated concentration of HOCl or H_2_O_2_ was spotted directly onto the agar surface. The plates were incubated at 30°C for 48 h, and then the diameters of the zones of growth inhibition (halos) were measured and the plates were photographed. Paper discs, often employed in this type of disc diffusion halo assay for the application of a chemical onto the surface of an agar plate, were not used since we found that paper discs had differential effects on HOCl that altered the uniformity of the zones of growth inhibition. The assays were carried out in duplicate on at least three independent days. The average change in the zone of growth inhibition was then assessed for statistical significance by ANOVA using GraphPad Prism. The comparison between the wild-type and mutant strains was assessed using a Dunnett test.

### RNA-seq analysis

*C. albicans* cells were freshly grown on YPD medium and then were grown in minimal BYNB medium with dextrose. A liquid culture was grown at 30°C overnight to saturation, diluted, and then kept in log phase growth overnight at 30°C. The cultures were then adjusted to 0.1 × 10^7^ cells/ml and were grown until they reached 1 × 10^7^ cells/ml. Ten-milliliter aliquots of cells were then incubated in the presence or absence of the indicated concentration of H_2_O_2_, HOCl, or benzoquinone at 30°C for the indicated time. Cells were then quick chilled and washed with ice-cold water, and pellets were quick-frozen with liquid nitrogen and reserved for subsequent analysis.

The extraction of RNA, preparation of cDNA, and sequencing reactions were conducted at GENEWIZ, LLC. (South Plainfield, NJ, USA). The RNA was extracted from a frozen cell pellet of 10^8^ cells using an RNeasy Plus Universal mini kit following the manufacturer’s instructions (Qiagen, Germantown, MD, USA). The RNA samples were quantified using a Qubit 2.0 fluorometer (Life Technologies), and RNA integrity was assessed using an Agilent TapeStation 4200 (Agilent Technologies, Santa Clara, CA, USA). RNA samples were prepared for sequencing using the NEBNext Ultra II RNA Library Prep Kit for Illumina following the instructions of the manufacturer (NEB, Ipswich, MA, USA). Briefly, samples were first enriched for mRNA using Oligo(dT) beads. The mRNA samples were then fragmented for 15 min at 94°C and then used as a template for cDNA synthesis. The ends of the cDNA fragments were repaired and then adenylated at 3′ ends. Universal adapters were ligated to the cDNA, followed by index addition and library enrichment by limited-cycle PCR. The sequencing libraries were then validated using an Agilent TapeStation (Agilent Technologies). They were then quantified with a Qubit 2.0 fluorometer (Invitrogen) and by quantitative PCR (KAPA Biosystems). The sequencing libraries were pooled, clustered on one lane of a flowcell, and then loaded on an Illumina HiSeq instrument (4,000 or equivalent) according to the manufacturer’s instructions and sequenced using a 2 × 150 bp paired end configuration. Image analysis and base calling of the data were conducted using HiSeq Control Software. The raw sequence data .bcl files generated from the Illumina HiSeq were converted into fastq files and de-multiplexed using Illumina’s bcl2fastq 2.17 software. One mismatch was allowed for index sequence identification.

Sequence data were then subjected to quality profiling, adapter trimming, read filtering, and base correction for raw data using fastp, an all-in-one FASTQ preprocessor (58). The high-quality paired-end reads were mapped to the *C. albicans* SC5314 genome (Candida Genome Database; Assembly 22) using HISAT2 (59). The read alignments obtained in the previous step were assembled with StringTie (60) and used to estimate transcript abundances. The absolute mRNA abundance of the samples was expressed as fragments per kilobase of transcript per million mapped reads. Analysis of differential gene expression was conducted using the DESeq2 (60) package (61) from Bioconductor (62) on R. GO term analysis was carried out at the ShinyGO web site (http://bioinformatics.sdstate.edu/go) (63).

## Data Availability

The RNA-seq data are freely available as Table S1 and have been deposited with the Sequence Read Archive of the National Library of Medicine of the National Institutes of Health under BioProject accession number PRJNA1013166.

## SUPPLEMENTAL MATERIAL

Table S1 mbio.02671-23-s0001.xlsx RNAseq analysis of *C. albicans* exposed to different types of oxidative stress.

Table S2 mbio.02671-23-s0002.docx Oligonucleotides used in this study.

ASM does not own the copyrights to Supplemental Material that may be linked to, or accessed through, an article. The authors have granted ASM a non-exclusive, world-wide license to publish the Supplemental Material files. Please contact the corresponding author directly for reuse.
