# Supplementary material for: Candida albicans resistance to hypochlorous acid
Source: mBio. 2023 Nov 30;14(6):e02671-23. doi: 10.1128/mbio.02671-23 (PMC10746268; doi:10.1128/mbio.02671-23)
Supplement: Table S2 — Oligonucleotides used in this study. [file mbio.02671-23-s0002.docx]

**TRX1_SNR52/R**

AGCTGCTATTAAACAAGCTTCAAATTAAAAATAGTTTACGCAAGTC

**TRX1_sgRNA/F**

AAGCTTGTTTAATAGCAGCTGTTTTAGAGCTAGAAATAGCAAGTTAAA

**TRX1-F**

TGTTTTTTCACCAACAAAATAGATATCAATTCTATTCAACCCTTTAACACTTAACACATAGTTATATTTTTTTTCAAAGACCAGTGTGATGGATATCTGC

**TRX1-R**

AGTTATTCCATTCAATTGTAAATATACACCCATCATCATCAATAATACAATCATCCAAAACTATTATTAGACTATCTTCAGCTCGGATCCACTAGTAACG

**TRX1 – Det-F**

CACGATCCATTACTAACC

**TRX1-Det-R**

AATAGTTACTTCGGCTGG

**TRX1-Det-Int-R**

ACGATTGACTTCTTCACC

**TRX1-Comp-F**

TCCCTTTAGTGAGGGTTAATTTCGAGCTTGGCGTAATCATGGTCATAGCTGTTTCCTGTGTGAAATTGTTATCCGCTCACTGACCAAGACAACTTGTTGG

**TRX1-Comp-R**

TCATATTGTCTTACTACTTTCTCTCCTTCATCGTCCTTCCGGCCCCCCCTCGAGGTCGACGGTATCGATAAGCTTGATATTACATTTCGGACATGACTTGGAAGTC

**SRX1_SNR52/R**

GTGGAATTGGTCTTTTGATTCAAATTAAAAATAGTTTACGCAAGTC

**SRX1_sgRNA/F**

AATCAAAAGACCAATTCCACGTTTTAGAGCTAGAAATAGCAAGTTAAA

**SRX1-F**

TTTTGAATACAACAATATTAGGTTTACTCATTCAAAGCATCAACCAGTTCAAGATAGATTAGTATTATAAACGTTCAAAACCAGTGTGATGGATATCTGC

**SRX1-R**

TTTAATTTTACACAAGAATGCATAGTGGTAACTGAATTAGTATCACTCCTAAACAGGGTTACAAATAACATCCATTCTGAGCTCGGATCCACTAGTAACG

**SRX1 – Det-F**

CATGCCTGACAAATCAGC

**SRX1-Det-R**

ATGCTGTATCTGATCATCC

**SRX1-Det-Int-R**

TGACTTTCTAGTTGCTGG

**SRX1-Int-R2**

CACACTATCAAAGAGAGC

**SRX1-Comp-F**

TCCCTTTAGTGAGGGTTAATTTCGAGCTTGGCGTAATCATGGTCATAGCTGTTTCCTGTGTGAAATTGTTATCCGCTCACGAAAAAGGAGGTTCATGACG

**SRX1-Comp-R**

TCATATTGTCTTACTACTTTCTCTCCTTCATCGTCCTTCCGGCCCCCCCTCGAGGTCGACGGTATCGATAAGCTTGATATATGTAAACAGAAGAACGGATACAAGG

**MXR1_SNR52-R3**

ACTTTAATGTCTACTAGACCCAAATTAAAAATAGTTTACGCAAGTC

**MXR1_sgRNA-F3**

GGTCTAGTAGACATTAAAGTGTTTTAGAGCTAGAAATAGCAAGTTAAA

**MXR1-F**

CACAGTTTTAGTTTCCTCGACCTTTCTTTCTTTCTTCATTTCTGTACTAGACATTTACAAATTCACCACTTGATTCAATAACCAGTGTGATGGATATCTGC

**MXR1-R**

TAATCCCATATTAACTATCGTATAGTAAACGCAGCAAAACTATATGCATTTCGGTTCTCTTCTTTTCTTATCTTCCCGCAAGCTCGGATCCACTAGTAACG

**MXR1- Det-F**

TGGCAATATCACGTGACC

**MXR1 - Det-R**

TTCATCTCCCACTGTAGC

**MRX1-Det-Int-F**

AGTAGACATTAAAGTCGG

**AYS1_sgRNA-F2**

GCAAACTTATGCTGCTATGGGTTTTAGAGCTAGAAATAGCAAGTTAAA

**AYS1_SNR52-R2**

CCATAGCAGCATAAGTTTGC CAAATTAAAAATAGTTTACGCAAGTC

**AYS1- Det-F**

TCAATGATTACGCAATGG

**AYS1-Det-R**

ATTTGCTCCTTATCGTCC

**AYS1-Det-Int-F**

TATACAGCTCCTCATTGG
